# The experience of social isolation in patients receiving peritoneal dialysis: a qualitative study

**DOI:** 10.1186/s40359-025-02367-y

**Published:** 2025-08-20

**Authors:** Keke Diao, Jiajia Wang, Yijia Huang, Yanjun Zhang, Dingshuo Guo, Luke Zhang, Yan Shan

**Affiliations:** 1 The third affiliated hospital of zhengzhou university, No.7 Rehabilitation Front Street, Erqi District, Zhengzhou City, Henan Province China; 2 School of Nursing and Health, Zhengzhou university, No. 101, Science Avenue, Gaoxin District, Zhengzhou city, Henan Province China

**Keywords:** Peritoneal dialysis, Patient experience, Qualitative study, Social isolation

## Abstract

**Background:**

Social isolation(SI), as a negative psychological state, can aggravate sleep disturbances and deterioration of renal function, seriously jeopardizing physical health, which is particularly prominent in peritoneal dialysis(PD) patients. However, most of the current research in this field is quantitative, and there are few qualitative studies on SI in PD patients. Understanding patients’ psychological experience of SI is essential for the targeted design of intervention programs. Therefore, the aim of this study is to investigate the experience of SI among PD patients.

**Methods:**

12 peritoneal dialysis patients who were hospitalized in a tertiary hospital in Zhengzhou City, Henan Province, were selected for the study using purposive sampling. Semi-structured in-depth interviews were conducted to collect data, and content analysis methods were used to analyze the data.

**Results:**

A total of 4 themes and 16 sub-themes were analyzed and extracted from this study, namely: (1) Objective manifestations of social isolation(Work isolation, Entertainment isolation, International isolation, Self isolation); (2) Subjective emotional feelings of social isolation. (Low self-esteem and sensitivity, Fear and concern, Solitude and loneliness, Dissembling and deception); (3) Multiple obstacles exacerbate the plight of social isolation(Shackles of over protection, Lack of self-worth, Agony of public misunderstanding, Burden of treatment expenditure, Limitation of treatment conditions); (4) Patients still crave multiple forms of social support(Appreciate Family support, Value Peer Support, Call for social support).

**Conclusion:**

This study provides insight into the experience of SI in PD patients and the reasons for it. SI in PD patients can manifest as Objective manifestations and Subjective emotional feelings that create significant psychological problems. The degree of SI is aggravated by multiple factors such as lack of self-worth and lack of public awareness. In the future, it is necessary to provide support to improve the SI problem through the collaboration of multiple fields such as family, peers and society.

**Supplementary Information:**

The online version contains supplementary material available at 10.1186/s40359-025-02367-y.

## Background

Chronic kidney disease (CKD) is now recognized as a major public health problem worldwide. The global prevalence of CKD is estimated at 13.4% (11.7–15.1%) [1]. In China, the prevalence of chronic kidney disease was 13.1%, and about 82 million people have been diagnosed with CKD [2, 3]. Along with the decline of renal function, CKD will evolve into end-stage renal disease(ESRD), and patients will need renal replacement therapy to maintain life. Studies showed that the number of patients receiving renal replacement therapy has exceeded 2.6 million [4] and is expected to reach 5.4 million by 2030 [5]. More than 500,000 patients have chosen dialysis as an alternative treatment, and more than 800,000 are expected by 2025 in China [6]. Peritoneal dialysis(PD) has been widely accepted by patients worldwide due to its simplicity, self-independence, and cost-effectiveness, and its utilization is increasing [7]. Unfortunately, PD treatments have a significant impact on patient’s daily lives and work, PD treatment restricts patients’ travel and reduces opportunities for job advancement [4]. These result in extremely limited social functioning.

Social isolation(SI) consists of five attributes: lack of interaction, self-isolation, loneliness, isolation, and meaninglessness, which can be reflected in the absence of social networks and the psycho-emotional aspects of loneliness [8, 9]. Studies have shown that SI disrupts an individual’s sleep patterns, exacerbates the risk of cardiovascular disease and deterioration of kidney function, and is even a major risk factor for increased mortality [10, 11], which is a danger to the physical and mental health of the patient. However, PD patients have prominent problems with SI. The prevalence of social functioning deficits in PD patients is 70%, with varying degrees of impairment in hyperactivity within the family, social activity outside the family, and social withdrawal [12]. In a qualitative study, it was shown that PD patients experienced a change in their social support structure and felt severe social abandonment and impaired social value [13]. In addition, PD patients had higher loneliness scores than patients with other chronic conditions, the reason may be the distance and time of multiple dialysis-limited socialising [14].

Existing research on patients’ SI is mostly quantitative, mainly surveying the current situation and analyzing the influencing factors, with less focus on patients’ emotional experiences and psychological states regarding SI. Only a few scholars have conducted qualitative interviews with older adults and cancer patients about SI [15]. To the best of our knowledge, this is the first qualitative study conducted on the experience of SI in PD patients. Therefore, the purpose of this study was to explore the experience of SI among PD patients and to provide referable values to alleviate the current situation of SI. The following research questions are posed:

Question 1: In what form does SI manifest itself in PD patients?

Question 2: What are the factors that aggravate as well as mitigate SI in PD patients?

Question 3: What help do patients need in order to alleviate SI?

## Methods

### Design

The study utilized a descriptive qualitative design and collected data through semi-structured in-depth interviews. Content analysis was used to analyze the data.

### Participants

We used a purposive sampling method to select PD patients hospitalized in the nephrology ward of a tertiary hospital in Zhengzhou City as participants. Inclusion criteria: (1) met the diagnostic criteria of K/DOQI guidelines and had been diagnosed with ESRD; (2) received PD treatment and had stable conditions; (3) age ≥ 18 years; (4) gave informed consent and voluntarily participated in this study. Exclusion criteria: (1) suffer from malignant tumor, cancer, or other serious organ diseases; (2) are unable to communicate normally or have serious cognitive disorders; (3) have been diagnosed with serious psychological disorders; (4) have changed renal replacement therapy, such as hemodialysis or renal transplantation. The sample size was based on the criterion that the interview data reached saturation and no new themes emerged, and a total of 12 PD patients were included in this study [16].

### Procedure

The nephrology ward of a tertiary hospital in Zhengzhou City was used as the recruitment site.

Face-to-face conversations were held between the researcher and the recruited participants in the ward, where the researcher introduced them to the purpose, methods and significance of the study. The researchers might answer the questions of the participants to build trust with the participants, as most of them were participating in the study for the first time. As far as possible, patients of different genders, ages, and marital statuses were selected for the study, so that the findings would be richer and the psychological experiences of the participants would be better understood. All participants met the inclusion criteria as well as signed a written informed consent.

### Data collection

The study followed the phenomenological research methodology in qualitative research by conducting face-to-face in-depth semi-structured interviews with patients who met the inclusion exclusion criteria. Patients had the right to terminate or end the interview at any time during the interview. The interviews were conducted for 20 to 40 min when the interviewee’s time and physical condition were suitable. The interview outline was initially developed after literature review and group discussion. Two PD patients were selected for pre-interviews (pre-interview data were not included in the analysis of results), and the interview outlines were revised after listening to the interviewees. After listening to the guidance of clinical nursing experts, psychologists and other professionals, the final interview outline was refined and formed. The outline of the interview is as follows: (1) Did you have any bad emotional and affective experiences when you knew you were going to be on dialysis (2)? What impact did these experiences have on your life and work (3)? Have you ever thought of staying away from social groups (e.g., family, friends, and coworkers, etc.) (4)? If you have these thoughts, what are the reasons (5)? Have your social activities changed after your dialysis? For example, how often and how you spend time with your family, friends and coworkers (6). What do you think are the reasons for these changes (7)? In the course of your social activities with people around you, what kind of help has been given to you by your family, friends, medical staff, and social organizations? What other areas do you need help with?

### Data analyse

Within 24 h after the interview, the researcher transcribed verbatim the audio recording of the entire interview process, including the verbal and non-verbal behaviors of the interviewee (e.g., frowning, sighing, crying, etc.). Information was organised according to the content of the notes recorded, numbered in the order in which the research subjects were interviewed. Content analysis was used to analyze the interview data, with coding derived directly from the data. Data were analysed using Nvivo software and supplemented with manual coding (1). The researcher carefully and thoughtfully read the interview data over and over again to familiarize herself with the data’s overall content; (2) analyze line by line, marking statements of significance for open-ended coding(*n* = 134); (3) compare and categorize similar or related codes, and gradually form themes and sub-themes; (4) define the themes, subthemes, and codes, and extract some representative examples from the data [17]. During the analysis process, the source material was reviewed several times to ensure correct interpretation.After initial analysis, we extracted a total of 160 meaningful sentences and 106 subthemes.

## Results

This study included 12 patients, a total of 6 males and 6 females, whose ages ranged from 19 to 69 years old, and other specific information is shown in Table 1.

**Table 1:** Demographic characteristics of the participants

Participants	Gender	Age	Marriage	Education	Time of diagnosis of disease	Age on dialysis
1	Male	45	Married	Master	5 years	3 years
2	Male	34	Unmarried	High school	1 year	6 months
3	Male	55	Married	College	5 years	4 years
4	Female	31	Married	Middle school	1 year	4 months
5	Female	29	Unmarried	High school	2 years	2 months
6	Male	25	Unmarried	College	10 months	3 months
7	Female	59	Married	Primary school	5 years	4 years
8	Male	48	Married	College	2 years	6 months
9	Female	69	Widowed	Primary school	1 year	1 month
10	Male	44	Divorced	Middle school	10 years	8 years
11	Female	60	Married	College	4 years	1 year
12	Female	19	Unmarried	High school	6 months	1 month

A total of 4 themes and 16 sub-themes were analyzed and extracted from this study: (1) Objective manifestations of SI(Work isolation, Entertainment isolation, International isolation,

Self isolation); (2) Subjective emotional feelings of SI(Low self-esteem and sensitivity, Fear and concern, Solitude and loneliness, Dissembling and deception); (3) Multiple obstacles exacerbate the plight of SI(Shackles of over protection, Lack of self-worth, Agony of public misunderstanding, Burden of treatment expenditure, Limitation of treatment conditions); (4) Patients still crave multiple forms of social support(Appreciate Family support, Value Peer Support, Call for social support).

### Objective manifestations of SI

#### Work isolation

#### Low self-esteem and sensitivity

The uremic face and the exposure to dialysis tubing change the patient’s body shape, which will attract the attention of others, unlike the past self and the general public, “peculiarities” that make the patient low self-esteem and sensitivity.

*“I didn’t feel much in the hospital*,* but once I was walking down the road and the kid looked at me and blurted out*,* ‘Mommy*,* this auntie’s tummy will pee*,*’ and I couldn’t wait to find a crack in the ground*,* and when I came back*,* I cried all the time and couldn’t see anyone.”(P5)*.

*“I used to be cute and beautiful*,* now I’m so dark and skinny I can’t cover my tubes*,* so how do I have the face to look in the mirror.”(P12)*.

#### Fear and concern

The patient’s fear and concern are reflected in the unknown nature of the disease process and the inability to move freely.

*“I’m still so young*,* this disease won’t be cured*,* a lifetime of dialysis*,* a lifetime*,* how many years do I have left to live ……”(P12)*.

Some patients reported that the management of long-term dietary control could be easily disrupted, which undermined confidence in overcoming the disease.

*“I have been on dialysis for several years*,* and the doctor always told me that I can’t eat this or that*,* the previous control can be good*,* which came back for a review*,* phosphorus or potassium how to be high again*,* the doctor said that I messed up the food*,* I don’t know what to do.”(P7)*.

#### Self-isolation and avoidance

Some patients said they preferred to stay in a closed environment, digesting their emotions on their own and not communicating with the outside world.

*“The first few days of dialysis*,* I cried every day*,* secretly*,* all the time*,* burrowed in the attic*,* couldn’t sleep at all at night*,* and in the morning my pillow was wet at dawn*,* and I didn’t want to talk to anyone.”(P4)*.

Some patients claimed that too much activity with others brought them negative emotions.

*“There are always guests who come to visit me*,* and I have to prepare wine and dishes to serve*,* and I can’t drink or eat*,* and I have to explain to them repeatedly about my illness*,* and I’m in pain.”(P3)*.

#### Alienated by others

For young unmarried patients, sudden bad news may lead to worries about the future life of the marriage partner and hinder the marriage process to some extent.

*“My wife ran away*,* was all set to get married*,* as soon as she heard I had this disease*,* her mom said nothing to marry*,* alas.”(P2)*.

Multiple daily dialysis fluid changes take up a great deal of the PD patient’s time, and the burdensome task interferes with travel and social interactions.

*“Decades of buddies*,* from childhood to growing up*,* do not contact. About dinner and drinks*,* can not eat high potassium*,* high phosphorus food*,* can not drink*,* said I am not cool; just play for two or three hours*,* have to go back to dialysis*,* slowly once or twice will not even call you.”(P10)*.

### Patients struggle to escape the effects of SI

#### Increasing cognitive and behavioral management

Some of the interviewees reported that knowledge about renal disease and PD was unfamiliar territory for them, and that self-confidence and satisfaction grew as they continued to make progress.

*“I didn’t even know what dialysis was before*,* I’d never even heard of it*,* and look at me now*,* I can change my own fluids*,* and I even taught that little girl yesterday.”(P9)*.

*“I now change the fluid three times a day*,* if the unit temporarily overtime something*,* I will time to slightly move back a little*,* and it does not matter.”(P1)*.

#### Seeking support from within the family

Patients reported that their work status changed after dialysis, their career plans were broken, the value of their work was not reflected, and the company of their family was important to them.

*“I have been a senior class teacher for more than ten years. After I got sick*,* the organization transferred me to the publicity and development department*,* the work content is very monotonous*,* I can’t go out to study and further training*,* my body doesn’t allow it (sigh). Fortunately*,* my wife has been with me*,* she has always told me that the body is the most important.”(P8)*.

*“I have two other children*,* the bigger can read well*,* the second also quite understand*,* the family is not in good condition (crying)*,* I now live on the children.”(P10)*.

#### Compromise and growth

The patients underwent a reorganization of the mind and saw both sides of the coin, thus adjusting his personal state, recognizing the goodness of life, and reintegrating into society.

*“After retirement*,* I can finally take a break*,* and it’s good to raise flowers and skate birds every day. It’s healthy for older people to eat lighter*,* learn about nutrition*,* and communicate more with people in our circle of patients.”(P11)*.

The patients determined that they would harmonize life and the disease to bring the two into balance.

*“I’m going back to school to continue my studies*,* applied for a separate dormitory room with the school to facilitate my dialysis*,* and for dialysis I’ll treat it as a break between classes to give myself a buffer and not get too tired.”(P6)*.

#### Reinventing the value of life

Some patients indicated a change in their previous perceptions and a greater emphasis on health.

*“When I was young*,* I worked hard to earn money*,* I socialized every day*,* I had all kinds of medicines in my drawer*,* and I couldn’t eat them all*,* so it’s meaningless to earn more money without a good body.”(P2)*.

Some patients also mentioned that through this treatment, they felt more deeply the love of family and the help of others, and that they were grateful and cherished the present moment.

*“This hospitalization children and his wife has been with*,* I am quite content*,* do not compare with others*,* contentment is always happy. God still favors me.”(P11)*.

### Multiple obstacles exacerbate the plight of SI

#### Shackles of over protection

Patients wanted their family and classmates to treat them as ordinary people, and too much interference could add to the burden of self-burden, and it was important for them to be “just like everyone else”.

“My mom won’t let me do anything, brings me tea, waits for me at the door even when I go to the bathroom, I don’t need her to do that to me, I’m not an invalid.”*(P12)*.

*“I want my classmates to treat me like a regular classmate and not take care of me too much*,* otherwise I will be constantly reminded that I am a sick person and can’t live a normal life without the care of others.”(P6)*.

#### Agony of public misunderstanding

Patients showed that malicious comments and rejection of patients aggravated the bad mood of patients’ worries, also resulted in the intentional segregation of others.

“In the village, not many people have seen this disease, and when they see my tubes, they ask questions and look at me in an annoying way.”*(P9)*.

*“I had a fight with that woman at the market*,* and she said I was sick to give way and looked down on me.”(P7)*.

#### Burden of treatment expenditure

Patients claimed that due to the disease they were much less physically active and even lost their old jobs.

*“The job is gone. I used to work as a door and window fabricator*,* and after I got sick*,* I couldn’t do it anymore*,* and my family’s money went to my doctor’s care ……”(P10)*.

*“I used to work in the field*,* raising pigs and chickens*,* but now I can’t work at all because of the swelling of my body*,* and it’s good to be able to cook. Look at this box of medicine*,* more than two hundred dollars*,* how much land must be planted ah …….”(P7)*.

#### Deficiencies in support systems

Patients reported it was difficult for them to obtain professional and effective support in a timely manner, and it was unable to accurately judge the status of their illnesses and choose how to seek medical treatment.

“I can only see the doctor once a day, and only briefly say a few words and then leave, although the patients will also talk to each other about the situation, we are always blindly guessing among us, or with the doctor to talk more peace of mind. The village doctor can’t help me at all, he doesn’t know about dialysis, he can only treat a cold or fever, and his medical skills are not as good as those of the doctors in the big hospitals.”*(P4)*.

The patients hoped that the society could give some care and help employment to improve the economic situation.

*“I can’t find a job to do*,* restaurant waiters*,* supermarket cashiers don’t want me*,* it’s because I have a disease*,* the boss is afraid to take the responsibility.”(P5)*.

## Discussion

The results of the present study identified the experience of SI in PD patients, i.e., the patients have a very serious problem of SI, and despite their efforts to get out of this state, they are still hindered.

SI in PD patients can appear through self-emotional experiences as well as manifestations of social interaction status. The patients in this study typically generally had a negative emotional state, with low self-esteem and sensitivity towards their current situation, while still having fear and concern about the future.This is consistent with previous studies [18]. After dialysis treatment, patients experienced a significant change in their own life, with a loss of free activities and a restricted life. Restlessness flourishes during the transition to a restricted life [19]. While undergoing fluid replacement therapy, patients with PD fear physical harm from complications such as peritonitis. At the same time, they become vulnerable and fearful in the face of endless treatments, thus worrying about the future. Several previous studies have confirmed the production of negative emotions [20, 21]. Prolonged dialysis occupies the patients’ time, requiring them to give up their hobbies, and social activities change accordingly. Patients must make adjustments when there are dietary and fluid restrictions [22]. To avoid eating treats with others, patients consciously reduce their outputs. Patients either actively or passively experience a decrease in social activities.

The patient struggles to get out of the SI dilemma. Health education helps patients learn about the disease, improve their self-management ability, resulting in positive emotions, such as sense of control and self-efficacy. The integration of emerging technologies with nursing interventions has been a trend in recent years due to the development of mobile web technologies. Pinto et al. [23]developed an application called NefroPortátil to assess and monitor the dietary intake of patients for the purpose of controlling the fluid and food intake of patients. The results showed that the application, in addition to favoring the nutritional control, improved the degree of perception of self-care of patients on dialysis with chronic renal failure. Besides, different forms of interventions such as web-based portal, remote patient monitoring, etc. are used in the areas of disease monitoring, health management, web-based diagnosis and treatment, and appointment booking for dialysis patients [24]. Future health management for PD patients will require mobile health as an integral part.

In China, PD patients tend to rely on their families as a unit of survival, living together as well as making joint decisions, when faced with major decisions such as dialysis [25]. Family members often take on the role of the listener and carer, using the family strengths resource to meet the patient’s physiological and psychological needs. The support of family members will bring more spiritual comfort to the patient.

Patients with ESRD realize that their health is irreversible, and they are nearing the end of their lives when they need replacement therapy to sustain them. First, the patient perceives the severity of the disease and the necessity of treatment to save the end of life [26]. The existential shift in consciousness here includes new thoughts, feelings, and actions as well as reflections on experiences [27]. Afterward, patients have to deal with the practical changes that come with dialysis, including mobility limitations and lifestyle changes, which is an inevitable part of the process for every PD patient. Many patients will change their concept of health in the final stages. They begin to focus on the present moment and the beauty of life.

Patients then increase their sense of value in life in behavioural health management [19, 28].

PD patients cannot escape SI due to inappropriate treatment, financial burdens and deficiencies in support systems. Patients will display abnormal behaviors such as fluid changes and dialysis line exposure in their social life, which will draw different attention from others. And the collapse of social empathy establishes a particular form in the patient’s relationship with others that fundamentally alters one’s subjectivity [29]. They attribute differences to the population of PD patients from us, resulting in social stereotypes, prejudice, and special treatment of patients [30, 31]. The patients feel shame as a result of this specialty, which contradicts their desire to integrate back into society and worsens social isolation. The patient’s socialization, lack of solo autonomy, and deterioration of social interactions has been limited by family’s over protection. This has been confirmed in previous study [32].

 In China, the economic benefits of PD are greater compared to hemodialysis, with an average annual medical cost of $12,841, which is 82.03% of the cost of hemodialysis [33, 34]. Almost all patients state that dialysis treatment is a significant financial burden on them. Consistent results have been reported from other countries [35–37]. Therefore, the State and the Government should optimise the healthcare system. For example, they should expand the age range for insurance coverage and adjust the reimbursement rate for patients to reduce their financial burden.

## Limitations

There are several limitations in this study. The findings in this study can’t be generalized to all PD patients of different ethnicities in other provinces since the patients were all from one province. In addition, only one interview was conducted with patients in this study.

In the future, multiple interviews will be conducted based on the changes in patients’ psychological experiences at different time periods after dialysis. This will improve the scientific validity of the study results.

## Conclusion

This study provides an understanding of the experience of SI in PD patients and the reasons for it. The results show that the SI of PD patients is a very serious problem that needs to be solved urgently, facing many problems such as heavy financial burden and public misunderstanding. In this regard, healthcare professionals should pay full attention to the real feelings of patients. Enhance the positive feelings of patients through interventions. In addition, with the support of the family and the social system, the patient’s social integration should be promoted so as to reduce the SI.

## Electronic supplementary material

Below is the link to the electronic supplementary material.

Supplementary Material 1

## Data Availability

The datasets generated and analyzed during the current study are not publicly available due to confidentiality and privacy related concerns, but are available from the corresponding author on reasonable request.

## Abbreviations

SI: Social isolation
PD: Peritoneal dialysis
