# Supplementary material for: The experience of social isolation in patients receiving peritoneal dialysis: a qualitative study
Source: BMC Psychol. 2025 Aug 20;13:947. doi: 10.1186/s40359-025-02367-y (PMC12366228; doi:10.1186/s40359-025-02367-y)
Supplement: Supplementary file 1 — Supplementary Material 1 [file 40359_2025_2367_MOESM1_ESM.doc]

**Supplementary Materials**

**1.Patient Informed Consent**

**Patient Informed Consent**

Dear friend

You have been invited to participate in a qualitative study on the experience of social isolation in peritoneal dialysis patients, which aims to improve social isolation, social participation and quality of life of peritoneal dialysis patients. You are invited to participate in this study because you meet the inclusion criteria of this study. Participation in this study will help improve negative emotions, promote socialization, and promote physical and mental health. If you agree to participate in this study, you will be asked to do the following:(i) a general information questionnaire to be completed; and (ii) an interview, which takes about 30 minutes and is audio-recorded throughout.

Special Note: ①This study is based on the review of a large number of medical literature distilled and summarized, scientific; ②If it brings you time inconvenience or physical discomfort, you have the freedom to withdraw from this study, which is your right; ③We will ensure your safety throughout the entire study; ④Participation in the study of all personal information, we will be your confidentiality, and will not be leaked out.

If you agree to participate, please sign the informed consent section below.

We sincerely look forward to your participation, thank you!

I have read the above, the researcher has explained this study to me, and I know that I can choose to participate or not.

I am volunteering to participate in this study.

**Signature of Participant ___________________**

**Date ___________________________**

Day/month/year

**2.Interview guide**

**Interviewer:**

I will be asking you some questions about your psychological experience and life changes after peritoneal dialysis. You may think that peritoneal dialysis has caused you a lot of problems and that it is difficult for you to get over it and be positive about your life. We are trying to understand the psychological experience of our patients so that we can help people through this difficult time. If there are any questions that are not clear, please let me know and I will ask them in a different way. Once I've asked you the question, feel free to answer, before we begin, do you have any questions?

**The outline of the interview is as follows:**

(1) Did you have any bad emotional and affective experiences when you knew you were going to be on dialysis?

(2) What impact did these experiences have on your life and work? Are you having trouble at work, affected by illnesses, or have a lack of motivation?

(3) Have you ever thought of staying away from social groups (e.g., family, friends, and coworkers, etc.)? Have you ever wanted to turn down a coworker party? Have you had times when you didn't want to go out with family or friends? Do you think these are related to your need for dialysis?

(4) If you have these thoughts, what are the reasons?

(5) Have your social activities changed after your dialysis? For example, how often and how you spend time with your family, friends and coworkers.Have you been hanging out less? Is it still the same group of people? Has the crowd changed?

(6) What do you think are the reasons for these changes?

(7) In the course of your social activities with people around you, what kind of help has been given to you by your family, friends, medical staff, and social organizations? What other areas do you need help with?

I don't have any more questions, do you have anything else to add that hasn't been mentioned?
